# Preoperative echocardiography and anesthetic drugs as predictors of post-induction hypotension during general anesthesia: a prospective observational study

**DOI:** 10.1038/s41598-024-76279-z

**Published:** 2024-10-28

**Authors:** Kyongsuk Son, Kentaroh Tarao, Masao Daimon, Tomoaki Yoshii, Atsushi Nakagomi, Maiko Hasegawa-Moriyama

**Affiliations:** 1 Department of Anesthesiology, Graduate School of Medicine, Chiba University, 1-8-1 Inohana-cho, Chuo-ku, Chiba, 260–8670 Japan; 2 Department of Anesthesiology, Chiba University Hospital, Chiba, Japan; 3 Department of Social Preventive Medical Sciences, Centre for Preventive Medical Sciences, Chiba University, Chiba, Japan; 4 Department of Cardiovascular Medicine, Chiba University Hospital, Chiba, Japan; 5 Department of Cardiovascular Medicine, International University of Health and Welfare, Mita Hospital, Tokyo, Japan

**Keywords:** Hypotension, General anesthesia, Sevoflurane, Norepinephrine, Cardiovascular diseases, Echocardiography

## Abstract

Post-induction hypotension (PIH) during general anesthesia is a serious concern due to its high incidence and association with adverse postoperative outcomes and prognosis. We prospectively enrolled consecutive 1,603 patients scheduled for non-cardiac and cardiac surgery under general anesthesia. The primary outcome was defined as the lowest mean blood pressure from induction of general anesthesia to the start of surgery: post-induction blood pressure (PIB). Multivariable regression analysis was used to investigate the relationships between PIB and preoperative echocardiographic measurements, types and doses of anesthetic agents, and preoperative comorbidities and oral medication. Left ventricle regional wall motion abnormality (RWMA) and lower estimated glomerular filtration rate significantly associated lower PIB. Factors significantly associated with higher PIB were high BMI, beta blockers, high pre-induction mean blood pressure, and starting induction in the afternoon. Sevoflurane and prophylactic norepinephrine use were associated with higher PIB as anesthetic interventions. Significant interactions were found between sevoflurane and RWMA (P for interaction: 0.01). This is the first study to demonstrate the feasibility of intervening in the selection of anesthetic agents based on echocardiographic findings and to recommend the use of sevoflurane and prophylactic norepinephrine for the prevention of PIH, especially in patients with RWMA.

**Supplementary Information:**

The online version contains supplementary material available at 10.1038/s41598-024-76279-z.

## Introduction

Regardless of the type of surgery, predicting and preventing complications in general anesthesia and performing it safely are crucial for improving postoperative outcomes. Perioperative hypotension is strongly linked to serious postoperative complications, including myocardial injury, stroke, and renal damage^1–4^. Post-induction hypotension (PIH) during general anesthesia, in particular, poses a significant concern due to its high incidence and its association with adverse postoperative outcomes and prognosis^5,6^. To date, various indicators have been proposed to predict the occurrence of PIH in previous studies, such as age, diabetes mellitus, preoperative inferior vena cava diameter^7–13^. However, most studies to date have been on a small scale or with limited parameters, and the predictors of PIH have not been fully elucidated.

We previously reported that assessment of a regional wall motion abnormality (RWMA) and E/e’ with preoperative transthoracic echocardiography might be helpful for stratification of patients at a risk of PIH in general anesthesia^13^. However, this study is a relatively small-scale retrospective study that only targeted surgical cases in which echocardiography was performed, and there is a possibility that case selection bias influenced the study results. In addition, previous studies on predicting PIH using echocardiography have limitations, such as using qualitative point of care ultrasound without quantitative echocardiographic parameters^14,15^ or not simultaneously considering the anesthesia methods^11,13^ in which most anesthetics have negative inotrope or vasodilatory effects that may cause hypotension^16,17^.

So far, preoperative echocardiography is not uniformly recommended for all patients undergoing non-cardiac surgery, especially in those deemed low-risk with maintained exercise tolerance. For these individuals, the necessity of delaying surgical procedures to conduct extensive cardiac evaluations is typically discouraged^18–21^. On the other hand, evidence suggests that echocardiography during the perioperative period may offer significant benefits in reducing complications among high-risk patients, as previous studies indicating an increase in postoperative complications in patients with echocardiographic abnormalities who also possess two or more risk factors according to the Revised Cardiac Risk Index (RCRI)^22,23^.

Thus, this study aimed to investigate prospectively whether the presence or absence of preoperative echocardiography (and the usefulness of its parameters if performed), anesthetic agents used, patient comorbidities, and preoperative medications can predict postoperative mean blood pressure (PIB) in a large number of consecutive patients undergoing surgery under general anesthesia.

## Methods

### Study population

This prospective, observational study was approved by the Clinical Ethics Committee of Chiba University (approval number: 3296, M10778). Informed consent was obtained from the study participants, including consent for anesthesia. Participants were enrolled in the study using an opt-out consent process. This approach was approved by the institutional review board as part of the study’s ethical considerations. Before patient enrolment, the trial was registered in the University Hospital Information Network (UMIN) Clinical Registry (Registration number: UMIN000037147, Principal investigator: Kyongsuk Son, Date of registration: June 24th, 2019, website: https://center6.umin.ac.jp/cgi-open-bin/ctr_e/ctr_view.cgi?recptno=R000042261). The procedures for this study on human subjects were in accordance with the Declaration of Helsinki and its later revisions.

**Figure 1:**
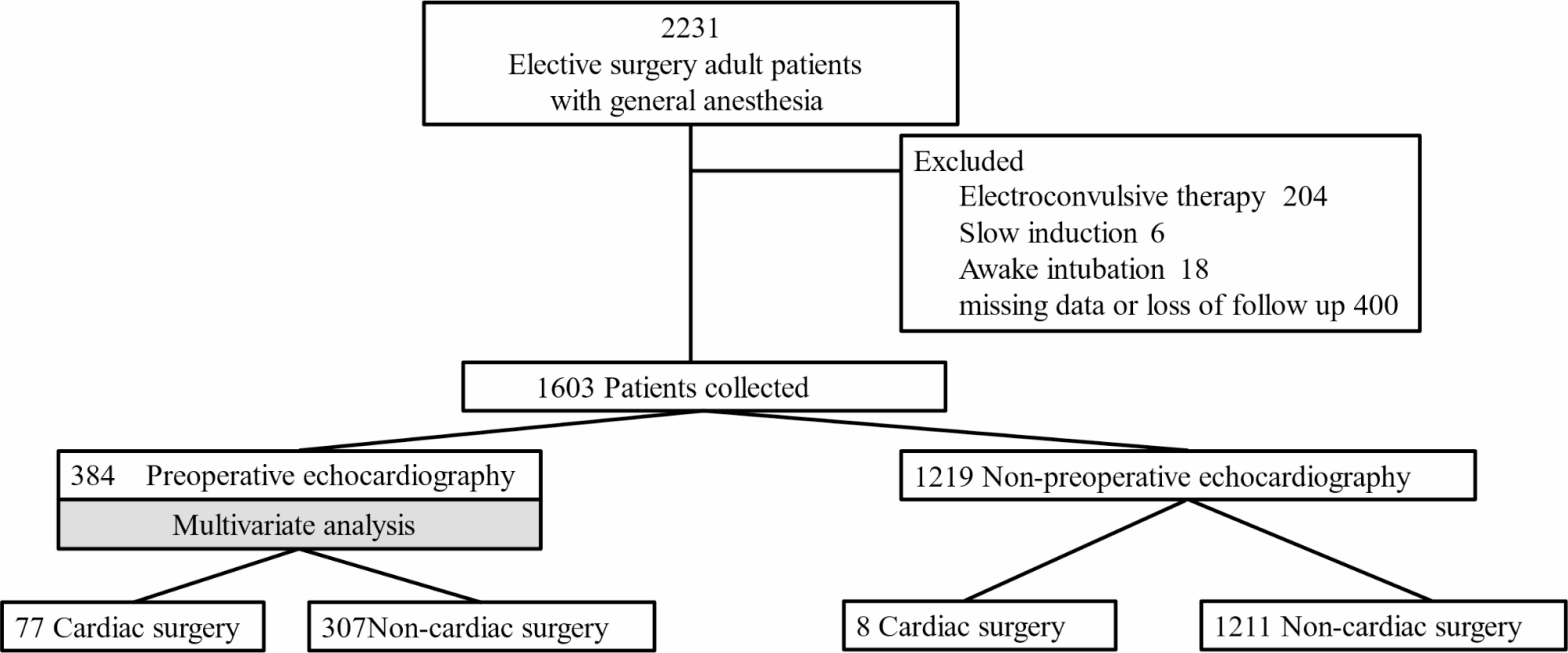
This figure illustrates the collection of elective surgery patients according to the specified inclusion and exclusion criteria. Multivariate analysis was performed on the subset of patients who underwent preoperative echocardiography, indicated by shading.

The article adheres to the applicable Strengthening the Reporting of Observational Studies in Epidemiology (STROBE) standards for observational studies^24^. We consecutively enrolled adult patients who underwent elective and non-cardiac surgeries under general anesthesia. The exclusion criteria were as follows:, (i) if the preoperative data sheet was not provided to the attending anesthesiologist, (ii) patients with missing information, iv) patients who had received emergency surgery and electroconvulsive therapy, and v) patients who received slow induction and awake intubation. Altogether, 2231 scheduled general anesthesia procedures were performed from August 2020 to May 2021, excluding patients who fulfilled the exclusion criteria. Finally, 1603 consecutive patients were enrolled in the study based on the eligibility criteria (Fig. 1).

### Data collection

For data collection, a data-sheet was distributed to the anesthesiologists before surgery (Tables 1 and 2). Preoperative oral angiotensin-converting-enzyme inhibitors (ACEIs), angiotensin II receptor blockers (ARBs), beta-blockers, calcium channel blockers, nicorandil, or nitroglycerin were continued or discontinued at the physician’s discretion. Moreover, the estimated glomerular filtration rate (eGFR) was calculated from serum creatinine using the approximate formula of the Japan Society of Nephrology [eGFR (mL·min-1·1.73 m^−2^)194 x Serum creatinine (-1.094) x Age(-0.287) x 0.739 (for women)^25^. History of coronary disease, heart failure, cerebral infarction, and cerebral hemorrhage were defined when identified in the questionnaire during a preoperative medical examination conducted by an anesthesiologist. Exercise tolerance was assessed using the metabolic equivalent of task (METs) by means of a medical questionnaire^18^. History of hypertension and dyslipidemia were defined when identified in the medical questionnaire or on oral medication use for these conditions. Atrial fibrillation was defined as the inclusion of its history in the medical questionnaire or a diagnosis of atrial fibrillation on an electrocardiogram.

### Anesthesia protocol

General anesthesia induction was achieved using either propofol or midazolam, supplemented with fentanyl and remifentanil, or an administration of rocuronium at a dosage of 0.6–1 mg/kg. Subsequent to tracheal intubation, the maintenance of anesthesia was facilitated through a regimen comprising sevoflurane, desflurane, and propofol in conjunction with remifentanil and fentanyl. Throughout the maintenance phase utilizing propofol, the bispectral index (BIS) was diligently monitored, aiming for a BIS value within the range of 40–60. In scenarios necessitating it, epidural anesthesia was implemented prior to general anesthesia induction, initiated with a 3 mL test dose of 1% lidocaine. Prophylactic administration of norepinephrine was characterized by its utilization preceding or concurrent with the induction of general anesthesia. Norepinephrine, prepared at a concentration of 20 mcg/mL, was administered via a peripheral intravenous route. The initiation dose of the continuous norepinephrine infusion was left to the discretion of the attending anesthesiologist. In instances where norepinephrine was not employed, bolus injections of ephedrine (4–8 mg/dose) or phenylephrine (0.05–0.1 mg/dose) were administered, with no data collected for the details of the dose.

The selection of the anesthesia method, vasopressor agents, and all other related decisions were left to the discretion of the attending anesthesiologist.

### Primary outcome

The primary outcome was delineated as the minimum mean blood pressure (MBP) recorded from the time of induction anesthetic administration to the commencement of surgical procedures^5,6^, hereby referred to as Post-induction blood pressure (PIB). Blood pressure measurement was conducted through non-invasive methods at 2–5 min intervals or via invasive techniques, contingent upon the patient’s clinical status or the nature of the surgical intervention. PIH was defined as the minimum MBP 65 mmHg or less from induction of general anesthesia to the start of surgery^26,27^.

### Echocardiography

Echocardiography was performed within 6 months prior to surgery for reasons such as history of cardiovascular disease, abnormal Electrocardiogram, advanced age, exercise tolerance less than 4METs or unknown, or prior to high-risk surgery^19,28^. Preoperative echocardiographic data were available to all anesthesiologists. The echocardiography lab operates according to the Japanese Society of Echocardiography’s standards^29^. Echocardiographic assessments were conducted in our dedicated laboratory, utilizing the using two cardiac ultrasound systems (Vivid E9®, GE Medical, Milwaukee, WI, USA; and Epic®, Philips, Andover, MA, USA). We disregarded echocardiograms from external sources, bedside evaluations, and transesophageal echocardiograms. The study encompassed patients who underwent quantification of cardiac chambers, focusing on the left ventricle (LV) diameter via 2D echocardiography as per the prevailing guidelines^30^. Measurements of the end-diastolic thickness for both the interventricular septum and the LV posterior wall were included. The LV ejection fraction (EF) was derived using the Teichholz method except in instances of LV RWMA, where LV volumes at end-diastole and end-systole were ascertained from apical views using the biplane disc summation method, with LVEF calculated by the equation: LVEF = 100 × (end-diastolic volume - end-systolic volume) / end-diastolic volume. Evaluation of diastolic function included analysis of mitral inflow velocities via pulsed Doppler, with the early (E) peak velocity from the mitral inflow pattern being noted. Pulsed tissue Doppler recorded mitral annular motion velocity at both lateral and medial annuli in the apical 4-chamber view, measuring the peak early diastolic velocity (e’) at the annulus and subsequently determining the E/e’ ratio^31^. Valvular disease severity was classified following existing guidelines^32,33^, with conditions of moderate severity or above deemed clinically significant. The estimated systolic pulmonary artery pressure was computed based on guidelines^34^, combining the tricuspid regurgitation gradient and estimated right atrial pressure from the inferior vena cava diameter. Pulmonary hypertension was defined as a tricuspid regurgitation pressure gradient of 36 mmHg or more^35^. For regional LV function assessment, a 17-segment model was employed, evaluating each segment independently across multiple views. LV reginal wall motion in each segment was scored as: (1) normal or hyperkinetic, (2) hypokinetic, (3) akinetic, and (4) dyskinetic. A regional wall motion abnormality was identified if any of the 17 segments was scored as hypokinetic, akinetic, or dyskinetic^30^.

### Statistical analysis

All statistical analyses were carried out using the R version 4.0.3 software (R Foundation for Statistical Computing, Vienna, Austria) and were reviewed by a statistical expert (A.N.). Parametric data are expressed as mean ± standard deviations (SDs) for continuous variables and as frequencies with percentages for categorical variables. Group comparisons between patients with and without TTE in non-cardiac surgery were performed using Student’s t-test for continuous variables and Fisher’s exact test for categorical variables.

Multivariable linear regression analysis was utilized to identify factors associated with PIB by incorporating input from a comprehensive literature review and expert opinions from anesthesiologists and cardiologists. The factors included the patient’s background clinical data (16 variables), preoperative medication (7 variables), anesthetic factors (6 variables), and echocardiographic parameters (7 variables) as explanatory variables. Regarding vasopressors, we compared cases where prophylactic administration of norepinephrine was used as an explanatory variable with those where only bolus administration was performed.

The required sample size was calculated using G*Power 3.1.9.7^36^, with an anticipated effect size f^2^ = 0.08, a significance level of 5%, and a power of 80%, a total of 357 cases were deemed necessary. The final sample size of 384 was considered sufficient for the analysis. (Tables 1, 2 and 3)

In the secondary analysis, we focused on the echocardiographic parameter RWMA, which was significantly associated with lower PIB in the primary analysis and previous study^13^. We included an interaction term between RWMA and type of maintenance anesthetic, as well as prophylactic use of norepinephrine, to explore their interaction effects on the association of RWMA with PIB. All *P* values were two-tailed. *P* values < 0.05 were considered statistically significant.

## Results

We performed an analysis on 1,603 patients scheduled for surgery, collecting data relevant to the study period. Echocardiography was conducted within 6 months prior to surgery in 384 patients (24.1%), of which 307 were non-cardiac surgery patients (20.2%). The incidence of PIH below MBP 65 mmHg occurred in 59.2%.

The incidence of PIH was significantly higher in the group with preoperative echocardiography (TTE group) compared to the group without preoperative echocardiography (non-TTE group) (65.6% vs. 57.2%, *P* = 0.004). In the TTE group, anesthesiologists with less than one year of experience were responsible for 13% of cases, and there was no significant difference in the incidence of hypotension between those with less than one year of experience and those with more. (*P* = 0.6)

The average time before surgery when echocardiography was done was 28[14–56] days for non-cardiac surgery patients. Age, prevalence of preoperative comorbidities and preoperative medications were significantly higher in patients undergoing echocardiography. (Tables 1, 2, 3 and 4)

**Table 1:** Patient characteristics and results of the univariate analysis for these variables.

	Total (*n* = 1603)	Preoperative TTE^#^ (*n* = 384)	Non Preoperative TTE^#^ (*n* = 1219)	Pvalue
*Sex (Male), n (%)	843(52.5)	241(62.7)	602(49.4)	<0.001
*Age, y	62.9 ± 5.62	71.5 ± 12.6	60.1 ± 15.6	<0.001
*Height, cm	161.2 ± 9.6	160.5 ± 10.4	161.4 ± 9.4	0.1
*BMI, kg/cm2	23.4 ± 4.2	23.6 ± 4.2	23.4 ± 4.2	0.26
*≦ 3 Mets, n (%)	33(2.1)	12(3.1)	21(1.7)	0.1
*Heart failure, n (%)	64(4)	50(13)	14(1.1)	<0.001
DM, n (%)	277(17.3)	97(25.3)	180(14.8)	<0.001
*CI, n (%)	67(4.2)	35(9.1)	32(2.6)	<0.001
*CH, n (%)	25(1.6)	7(1.8)	18(1.5)	0.64
*Hypertension, n (%)	714(44.5)	263(68.5)	451(37)	<0.001
*Dyslipidemia, n (%)	156(9.7)	58(15.1)	98(8)	<0.001
*AF, n (%)	83(5.2)	55(14.3)	28(2.3)	<0.001
RCRI ≧ 2, n(%)	80(5)	60(15.6)	20(1.6)	< 0.001

Data are expressed as mean ± SD or expressed as the number of patients (% for of total patients in each category). *TTE* transthoracic echocardiography, *BMI* body mass index, *Met* metabolic equivalent, *DM* Diabetes mellitus, *CI* Cerebral infarction, *CH* cerebral hemorrhage, *AF* Atrial fibrillation *, variables used for the logistic regression analysis. #, P values presented the results of univariate analysis in these patient groups

**Table 2:** Preoperative medications and conditions, and results of univariate analysis for these variables.

	Total (*n* = 1603)	preoperative TTE^#^ (*n* = 384)	Non preoperative TTE^#^ (*n* = 1219)	Pvalue
Preoperative medication				
*ACE, n (%)	48(3)	22(5.7)	27(2.2)	0.001
*ARB, n (%)	303(18.9)	106(27.6)	197(16.2)	< 0.001
*βblocker, n (%)	156(9.7)	89(23.2)	67(5.5)	<0.001
*CCB, n (%)	468(29.2)	158(41.1)	310(25.4)	<0.001
*Nicorandil, n (%)	26(1.6)	18(4.7)	8(0.7)	<0.001
*Nitroglycerin, n (%)	10(0.6)	8(2)	2(0.2)	< 0.001
*Insulin, n (%)	79(4.9)	36(9.3)	43(3.5)	<0.001
Preoperative condition				
*Starts of the induction in the afternoon, n (%)	532(33.2)	113(29.4)	419(34.4)	0.08
*Pre-induction MBP, mmHg	106 ± 14.9	104 ± 20.1	104 ± 17.3	0.1
*Hemoglobin, g/dl	13 ± 2	12.4 ± 2.1	13.2 ± 1.9	<0.001
*eGFR, ml/min/1.73cm2	60.5 ± 24	56.2 ± 23.6	61.8 ± 24	<0.001
*Current smoke, n (%)	46(2.9)	12(3.1)	34(2.8)	0.72

Data are expressed as mean ± SD or expressed as the number of patients (% for of total patients in each category). *ACEI* angiotensin-converting-enzyme inhibitor, *ARB* angiotensin II receptor blocker, *β* blocker, beta blocker, *CCB* calcium channel blocker, *Insulin* insulin use, *MBP* mean blood pressure, *eGFR* estimated glomerular filtration rate. *, variables used for the logistic regression analysis. #, P values presented the results of univariate analysis in these patient groups

**Table 3:** The anesthetics used for induction of anesthesia and results of the univariate analysis for these variables.

	Total (*n* = 1603)	Preoperative TTE ^#^(*n* = 384)	Non preoperative TTE^#^ (*n* = 1219)	Pvalue
*Propofol, mg/kg	1.25 ± 0.35	1.14 ± 0.34	1.27 ± 0.35	< 0.001
Midazolam mg	2.6 ± 0.9	2.6 ± 0.9	2.6 ± 1	NA
*Fentanyl, mcg/kg	1.75 ± 0.59	1.8 ± 0.69	1.73 ± 0.54	0.23
*Remifentanil, mcg/kg/min	0.18 ± 0.08	0.15 ± 0.07	0.18 ± 0.08	<0.001
*Sevoflurane, n (%)	214(13.3)	71(18.5)	143(11.7)	0.001
*Desflurane, n (%)	863(53.8)	197(51.3)	668(54.8)	0.24
Total venous anesthesia, n (%)	526(32.8)	117(30.5)	408(33.5)	0.29
*NE administration, n (%)	418(26.1)	192(50)	226(18.5)	<0.001
NE dose, mcg/kg/min	0.04 ± 0.019	0.04 ± 0.018	0.04 ± 0.02	NA

Data are expressed as mean ± SD or expressed as the number of patients (% for of total patients in each category). *TTE* transthoracic echocardiography, *NE* norepinephrine. *; variables used for the logistic regression analysis. #; P values presented the results of univariate analysis in these patient groups

**Table 4:** Baseline echocardiographic variables.

Parameter	*n* = 384
LVDd, mm	45.6 ± 7.68
LVPWTd, mm	9.34 ± 1.71
IVSTd, mm	9.52 ± 1 .87
*LVDd/BSA, mm/m^2^	27.3 ± 3.92
EF, %	61.6 ± 8.07
*EF 45% or less, n (%)	20(5.2)
E/A	0.85 ± 0.43
DcT, ms	229 ± 84.2
E/e’	11.9 ± 8.1
Estimated systolic PAP, mmHg	30 ± 11.3
Pulmonary hypertension, n (%)	31(8)
*RWMA, n (%)	62(16.1)
*AR ≥ moderate, n (%)	13(3.38)
*AS ≥ moderate, n (%)	51(13.3)
*MR ≥ moderate, n (%)	24(6.25)
*TR ≥moderate n (%)	15(3.9)

Data are expressed as mean ± SD or expressed as the number of patients (% for of total patients in each category). *LVDd* left ventricular diameter at end-diastole; LVPWTd, left ventricular posterior wall thickness at end-diastole, *IVSTd* interventricular septum thickness at end-diastole, *BSA* body surface area, *EF* ejection fraction, *DcT* deceleration time, *PAP* pulmonary artery pressure, *RWMA* regional wall motion abnormality, *AR* aortic regurgitation, *AS* aortic stenosis, *MR* mitral regurgitation, *TR* tricuspid regurgitation

However, there were no significant differences in exercise tolerance, with only 2.6% of echocardiography patients having exercise tolerance that could not be assessed or was less than 4 METs. The rate of abnormal echocardiographic findings in patients for whom echocardiography was not recommended based on RCRI index and exercise tolerance was 25.5%.

Out of the patients with pre-existing ischemic heart disease, 23/68 (35.4%) exhibited RWMA as observed through echocardiography, with an EF averaging 58.6 ± 12.1%. The rate of prophylactic norepinephrine use before induction was significantly higher in patients with RWMA compared with patients without RWMA (47.5% and 62.9%, *P* = 0.037), although there was no significant difference in norepinephrine dose. (with RWMA 0.04 ± 0.018 mcg/kg/min, without RWMA 0.04 ± 0.018 mcg/kg/min, *P* = 0.79) Of the patients with RWMA, 34% were cardiac surgery patients. PIH in non-cardiac surgery patients with RWMA was 39%, while PIH in cardiac surgery patients with RWMA was 28% (*P* = 0.576). Although the difference was not statistically significant, PIH tended to be less common in cardiac surgery.

In the multivariate analysis with preoperative echocardiography, RWMA was significantly associated with low PIB. Additionally, a lower eGFR was significantly related to a lower PIB. On the other hand, a high PIB was significantly associated with a high BMI, use of β-blockers, high pre-induction mean blood pressure, use of sevoflurane, prophylactic administration of norepinephrine, and starts of the induction in the afternoon (Table 5). A potentially significant interaction effect of sevoflurane and RWMA was shown in a multivariate analysis. In contrast, desflurane and TIVA had no interaction effects with RWMA. Furthermore, no interaction effects were shown for norepinephrine. (Table 6)

**Table 5:** Result of the multiple regression analysis with post-induction blood pressure.

	Coefficient	Lower 95%CI	Upper 95%CI	*p* value
Positive factor				
BMI	0.35	0.15	2.28	0.02
βblocker	3.26	0.09	6.43	0.04
Pre-induction MBP	0.15	0.04	3.71	< 0.001
sevoflurane	5.48	2.08	2.63	0.009
norepinephrine	3.19	1.35	2.38	0.02
Start in the afternoon	3.22	1.33	2.48	0.02
Negative factor				
RMWA	-4.52	1.95	-2.32	0.02
Lower eGFR	-0.05	-0.027	-2.07	0.04

*CI* confidence interval, *BMI* body mass index, *βblocker* beta blocker, *MBP* mean blood pressure, *RWMA* regional wall motion abnormality, *eGFR* estimated glomerular filtration rate

**Table 6:** Interaction effects between left ventricle regional wall motion abnormality and each factor.

	single	*p* value	interaction	*p* value
Sevoflurane	5.48	0.009	8.33	0.01
Desflurane	-2.76	0.23	-3.29	0.18
TIVA	N/A	N/A	-1.12	0.67
NE administration	3.3	0.02	-0.27	0.9

*TIVA* total intravenous anesthesia, *NE* norepinephrine

In the present study, a history of valvular disease above moderate was not associated with PIB. The estimated regression coefficients for each valvular disease were aortic stenosis − 2.1 (*P* = 0.3), aortic regurgitation − 4.4 (*P* = 0.2), mitral regurgitation 1.7 (*P* = 0.5), tricuspid regurgitation − 5.0 (*P* = 0.1), and all except mitral regurgitation tended to decrease although not significantly. The factors of RCRI other than ischemic heart disease without RWMA and renal function were not significantly associated with PIB. (Supplementary file)

## Discussion

In this study, we showed that the presence of RWMA in preoperative echocardiographic findings and preoperative low eGFR are significantly associated with low PIB. Despite a significantly higher rate of prophylactic norepinephrine use compared to those without RWMA, patients with RWMA still exhibited lower PIB. Additionally, an interaction between sevoflurane and RWMA was identified, indicating that sevoflurane may helps in maintaining blood pressure during induction of general anesthesia in patients with RWMA.

Previously, we reported that RWMA was a risk factor (Odds ratio 6.65, P< 0.01) for PIH ( MAP ≤50mmHg) in a retrospective study (19). In the present study, we found similar results in a larger, consecutive, prospective observational study. Other studies have shown that transthoracic echocardiography (TTE) models incorporating RWMA can effectively predict post-operative complications when used in conjunction with RCRI^22^. On the other hand, however, it has also been reported that echocardiographic indices such as low EF and RWMA alone do not enhance the prediction of postoperative complications in perioperative patients^37^. The potential of echocardiography to predict postoperative complications remains controversial, with one of the reasons being the lack of interventions to prevent post- and intra-operative hypotension according to echocardiographic findings^38^. The significant interaction between the presence of RWMA and the use of sevoflurane shown in this study suggests that sevoflurane may help prevent PIH in patients with RWMA. This is the first study, to our knowledge, to demonstrate the feasibility of intervening in the selection of anesthetic agents based on echocardiographic findings and, moreover, to recommend the use of sevoflurane for the prevention of PIH, especially in patients with RWMA.

In this study, we not only identified risk factors for low PIB but also explored factors contributing to the maintenance of PIB. Our findings suggest potential interventions for anesthesiologists to prevent PIH, including the prophylactic use of norepinephrine prior to the induction of general anesthesia and the use of sevoflurane. Previous research has shown that desflurane has a greater hypotensive effect compared to sevoflurane^39,40^, supporting our study findings that sevoflurane is significantly associated with higher PIB. Additionally, inhaled anesthetics have a pharmacological preconditioning effect that may be advantageous in patients with ischemic heart disease^41^. In combination with the results of the present study, this supports the use of sevoflurane in patients with ischemic heart disease, especially in those with RWMA.

Previous research has documented the impact of low-concentration norepinephrine, administered through a peripheral line concurrently with the induction agent, on intraoperative and postoperative blood pressure in non-cardiac surgery^42^. Similarly, our study found that prophylactic administration of norepinephrine before induction was significantly associated with higher PIB. While previous research has established blood pressure thresholds for intervention, the optimal initial dosage of norepinephrine has not been extensively explored. In this study, the frequency of norepinephrine use was significantly higher among patients with ischemic heart disease and RWMA, yet the initial doses administered showed no significant differences. Our findings suggest that patients presenting with RWMA on preoperative echocardiography or those with low eGFR should receive interventions that include an increased initial dose of norepinephrine.

Most general anesthetics have been found to have a hypotensive effect due to their vasodilatory and negative inotropic effects^10,17,43^. This is particularly prominent with propofol, whereas it has also been reported with other inhaled anesthetics^44^ and remifentanil^43^. Regarding the effects of anesthetics in cardiac patients, propofol has been reported to be associated with significant induction hypotension in patients with aortic stenosis compared with other intravenous anesthetics such as remimazolam or etomidate^45,46^. However, propofol was not a significant risk factor for low PIB in the present study, presumably because it is used in a large number of cases and is used at a titrated dose. Independent risk factors for postoperative cardiovascular complications related to valvular disease include moderate to severe mitral regurgitation and increased aortic valve pressure gradient^23^. In the present study, no effect on blood pressure was found in the presence of valvular disease, however, as the prevalence of either is not so high in this study, it is necessary to investigate this issue further in large study.

### Limitation

First, this study analyzed subjects whose echocardiography was performed up to six months before surgery, potentially not reflecting the circulatory dynamics immediately prior to anesthesia induction. Previous research has linked the diameter of the inferior vena cava^11,12^ with PIH, a condition that may be influenced by decreased circulating plasma volume. In our study, the median time from the echocardiographic assessment to surgery was approximately one month, which is distinct from the preoperative point of care ultrasound provided by focused cardiac ultrasound (FOCUS)^47^. While there are reports that FOCUS interventions can mitigate PIH^48^, no improvements in outcomes have been demonstrated with FOCUS alone^49,50^, and current practices do not solely rely on FOCUS without well-measured echocardiographic assessments. Therefore, the results of this study should be available for risk stratification separately from FOCUS or in combination with it.

Secondly, a limitation of this study is that it included cardiac surgery patients, which necessitates caution when generalizing the results. However, as anesthetic induction methods and patient background factors were considered in the analysis, and the endpoints were limited to blood pressure measurements after anesthetic induction prior to the start of surgery^5^, the influence of cardiac surgery on these results is likely to be minimal.

Thirdly, since this study is observational, a prospective randomized trial is necessary to investigate the causal relationship between anesthetic agents and PIB. Furthermore, the interaction between sevoflurane and cardiac function was not elucidated in this study. In addition, although right ventricular function data such as pulmonary artery pressure were not included in this analysis, comorbid pulmonary hypertension is likely to have a significant impact on the patient’s cardiac dynamics under general anesthesia^51^. Further studies focusing on the mechanisms of blood pressure variability due to inhaled anesthetics and right ventricular function are warranted.

## Conclusion

In patients who underwent preoperative echocardiography, echocardiography may be helpful for identifying patients at risk of low PIB, particularly with RWMA, thereby informing anesthetic management to reduce the risk of postoperative complications. Optimizing the use of preoperative echocardiography for blood pressure control after induction of general anesthesia may be advantageous. The strategic use of sevoflurane and prophylactic norepinephrine could be beneficial in managing patients with RWMA. Further research is needed to explore the causal relationship between patients with cardiac disease and anesthetics and to optimize perioperative treatment strategies.

## Supplementary information

Below is the link to the electronic supplementary material.

Supplementary Material 1

## Data Availability

The datasets generated and/or analyzed during the current study are not publicly available due [to maintain the privacy of the patients participating in the study] but are available from the corresponding author on reasonable request.

## Declarations

### Clinical Trial Details

The trial was registered in the UMIN (University Hospital Information Network) Clinical Registry on June 24th, 2019.

### Registration number

UMIN000037147. Registry URL: https://center6.umin.ac.jp/cgi-open-bin/ctr_e/ctr_view.cgi? recptno=R000042261.
