# Supplementary material for: Preoperative echocardiography and anesthetic drugs as predictors of post-induction hypotension during general anesthesia: a prospective observational study
Source: Sci Rep. 2024 Oct 28;14:25717. doi: 10.1038/s41598-024-76279-z (PMC11519647; doi:10.1038/s41598-024-76279-z)
Supplement: Supplementary file 1 — Supplementary Material 1 [file 41598_2024_76279_MOESM1_ESM.docx]

**Supplemental data.**

Result of the multiple regression analysis with post-induction blood pressure

|  | Coefficient | Lower 95%CI | Upper 95%CI | p value |
| --- | --- | --- | --- | --- |
| Sex | -2.33 | 1.95 | -1.21 | 0.23 |
| Age | -0.1 | 0.06 | -1.67 | 0.1 |
| Height | 0.09 | 0.1 | 0.93 | 0.35 |
| BMI | 0.35 | 0.15 | 2.28 | 0.02 |
| ≦３Mets | -0.72 | 3.5 | -0.21 | 0.84 |
| Heart failure | -1.28 | 2.06 | -0.62 | 0.54 |
| Cerebral infarction | 1.15 | 2.17 | 0.53 | 0.6 |
| Cerebral hemorrhage | 5.51 | 4.46 | 1.24 | 0.22 |
| Hypertension | 0.56 | 1.53 | 0.37 | 0.71 |
| Dyslipidemia | 1.3 | 1.65 | 0.79 | 0.43 |
| Atrial fibrillation | 1.61 | 1.92 | 0.84 | 0.4 |
| ACE | -3.5 | 2.71 | -1.28 | 0.2 |
| ARB | -1.66 | 1.46 | -1.14 | 0.26 |
| βblocker | 3.26 | 1.61 | 2 | 0.04 |
| Calcium channel blocker | 2.56 | 1.36 | 1.89 | 0.06 |
| Nicorandil | 1.87 | 3 | 0.64 | 0.53 |
| Nitroglycerin | -3.55 | 4.27 | -0.83 | 0.4 |
| Insulin use | -0.07 | 2.17 | -0.03 | 0.97 |
| Starts of the induction in the afternoon | 3.22 | 1.33 | 2.48 | 0.02 |
| Pre-induction MBP | 0.15 | 0.04 | 3.71 | <0.001 |
| Hemoglobin | -0.32 | 0.32 | -0.99 | 0.32 |
| Lower eGFR | -0.05 | -0.027 | -2.07 | 0.04 |
| Current smoke | 0.1 | 1.6 | 0.06 | 0.95 |
| Propofol | 2.57 | 1.56 | 1.65 | 0.1 |
| Fentanyl | -0.84 | 0.71 | -1.17 | 0.24 |
| Remifentanil | -10.4 | 8.1 | -1.3 | 0.2 |
| Sevoflurane | 5.48 | 2.08 | 2.63 | 0.009 |
| Desflurane | -2.76 | 2.07 | -1.33 | 0.18 |
| Norepinephrine administration | 3.19 | 1.35 | 2.38 | 0.02 |
| LVDd/BSA | 0.27 | 0.18 | 1.5 | 0.14 |
| EF 45% or less | 0.63 | 3.29 | 0.19 | 0.85 |
| RWMA | -4.52 | 1.95 | -2.32 | 0.02 |
| AR ≥moderate | -4.41 | 3.52 | -1.25 | 0.2 |
| AS ≥moderate | -2.07 | 2.06 | -1.01 | 0.32 |
| MR ≥moderate | 1.74 | 2.76 | 0.63 | 0.53 |
| TR　≥moderate | -4.96 | 2.22 | -1.54 | 0.12 |

CI, confidence interval; BMI, body mass index; βblocker, beta blocker; MBP, mean blood pressure; RWMA, regional wall motion abnormality; eGFR, estimated glomerular filtration rate; AR, aortic regurgitation; AS, aortic stenosis; MR, mitral regurgitation; TR, tricuspid regurgitation.
